# Oral bisphosphonate-related osteonecrosis of the jaws in rheumatoid arthritis patients: a critical discussion and two case reports

**DOI:** 10.1186/1746-160X-7-7

**Published:** 2011-04-27

**Authors:** Nicolau Conte-Neto, Alliny S Bastos, Luis C Spolidorio, Rosemary AC Marcantonio, Elcio Marcantonio

**Affiliations:** 1 UNESP - Univ. Estadual Paulista, School of Dentistry, Department of Diagnosis and Surgery, Division of Periodontology, Rua Humaitá, 1680, 14801-903 Araraquara, SP/Brazil; 2 UNESP - Univ. Estadual Paulista, School of Dentistry, Department of Physiology and, Pathology, Division of Pathology, Rua Humaitá, 1680, 14801-903 Araraquara, SP/Brazil

## Abstract

**Background:**

Bisphosphonate-related osteonecrosis of the jaw (BRONJ) is a clinical condition characterized by the presence of exposed bone in the maxillofacial region. Its pathogenesis is still undetermined, but may be associated with risk factors such as rheumatoid arthritis (RA). The aim of this paper is to report two unpublished cases of BRONJ in patients with RA and to conduct a literature review of similar clinical cases with a view to describe the main issues concerning these patients, including demographic characteristics and therapeutic approaches applied.

**Methods:**

Two case reports of BRONJ involving RA patients were discussed

**Results:**

Both patients were aging female taking alendronate for more than 3 years. Lesions were detected in stage II in posterior mandible with no clear trigger agent. The treatment applied consisted of antibiotics, oral rinses with chlorhexidine, drug discontinuation and surgical procedures. Complete healing of the lesions was achieved.

**Conclusions:**

This paper brings to light the necessity for rheumatologists to be aware of the potential risk to their patients of developing BRONJ and to work together with dentists for the prevention and early detection of the lesions. Although some features seem to link RA with oral BRONJ and act as synergistic effects, more studies should be developed to support the scientific bases for this hypothesis.

## Background

Bisphosphonates (BPs) are a class of drugs commonly prescribed for bone diseases due to their osteoclast inhibition property. This class of drugs has been widely used for osteoporosis and corticosteroid-induced osteoporosis in patients with rheumatoid arthritis (RA). However, reports of bone necrosis induced by bisphosphonates (BRONJ) have generated great concern regarding the side effects of these drugs. Although RA has been considered a risk factor for this kind of osteonecrosis [1,2], the relationship between these diseases has not, until now, been completely elucidated.

The aim of this paper is to report two unpublished cases of BRONJ in non-neoplastic patients with RA and to conduct a literature review of similar clinical cases with a view to describing the main issues related to these patients, including demographic characteristics and therapeutic approaches.

## Case 1

A 58-year-old woman presented herself at a private dental clinic in December, 2008, complaining about an intense spontaneous pain in the mandibular right side after a prosthesis replacement in an implant area that was installed sixteen years previously. The review of the patient's medical history revealed that she started a therapy with Fosamax^® ^(alendronate sodium) 70 mg, once a week for the treatment of rheumatoid arthritis in 2004. The patient had no history of smoking, radiotherapy, infectious process or trauma in the maxillo-facial region, and the dental implant presented normally until the symptoms began.

**Figure 1:**
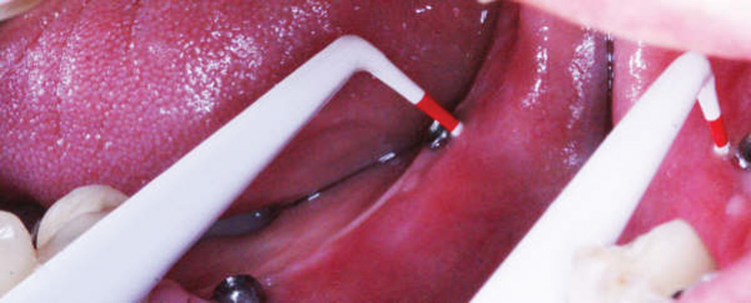
**Clinical aspect of the BRONJ lesion**. Mucosal erythema surrounding the distally right implant associated with an increase on probing depth values with no gingival recession or bone exposure.

**Figure 2:**
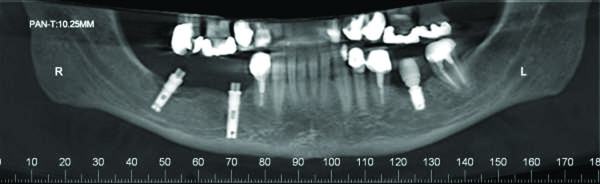
**Imaging aspect of the BRONJ lesion**. Computed tomography showing a radiolucency with aspect of loss of crestal bone around the right distally implant.

Upon clinical examination, a mild erythema was evident in the mucosa surrounding the distally right dental implant, without clinical evidence of purulent discharge, gingival recession or bone exposure. However, probing revealed increasing depth values and detachment of the mucosa from the periimplantar bone with biological seal loss was observed (Figure 1). A computed tomography (CT) was requested and showed a substantial radiolucency around the involved dental implant, featuring loss of the crestal bone. (Figure 2)

**Figure 3:**
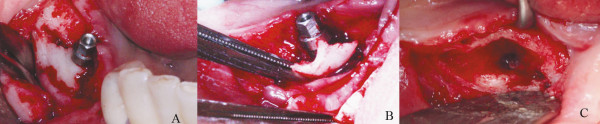
**Surgical approach of the BRONJ lesion**. A) Surgical exposition of the distally right implant showing a large bone sequestrum around the dental implant; B) Sequestrectomy of the bone necrosis around the dental implant; C) Surgical area after the debridement showing a bone bleeding surface associated with the dental implant removal.

Periimplantitis was the primary hypothesis considered at that time, but BRONJ was also considered. The initial treatment plan was mouth-rinsing with chlorhexidine 0.12% four times a day and antibiotic therapy with Clindamycin 300 mg twice a day for 10 days, since the patient had allergy for β-lactam antibiotics. Surgical decontamination of the implant surface was also planned; however, upon mucosal flap incision, there was no indication of any exposition of implant threads, but there was a large zone of necrotic bone forming a sequestrum area (Figure 3a). Therefore, it was opted to removal of the implant with sequestrectomy and debridement (Figure 3b) until a bleeding bone was observed (Figure 3c). An interrupted suture was made with 4-0 silk in an attempt to close the wound primarily without tension. After medical consensus, alendronate was suspended.

**Figure 4:**
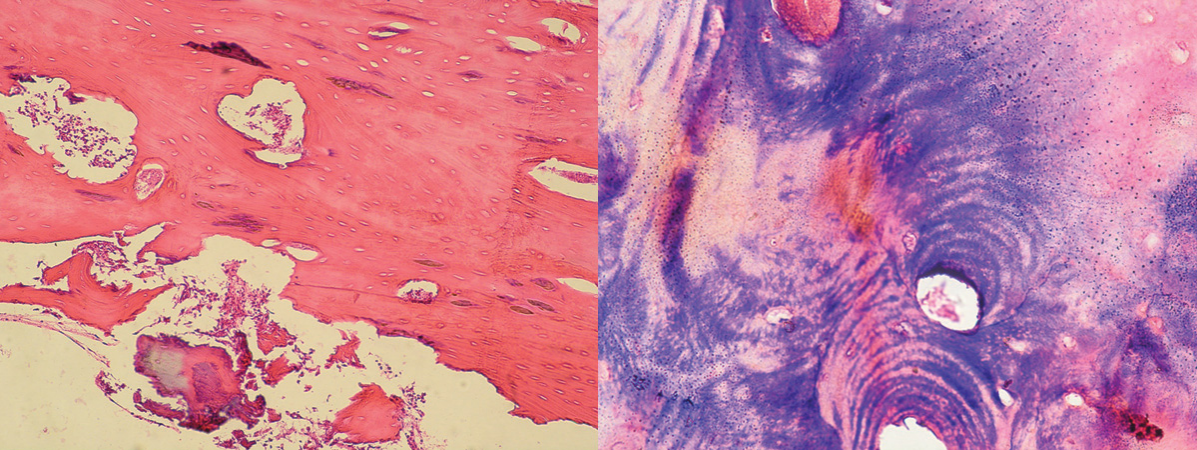
**Histological aspects of bone samples**. A) H & E stained section showing bone necrosis (Original magnification × 40); B) Gram stained section showing gram negative and positive bacteria (Original magnification × 100)

The bone specimen obtained was fixed, processed and paraffin embedded for histological analysis. Hematoxylin and eosin (H&E) staining was used for histological observation by light microscopy. The results revealed necrotic lamellar bone fragments with chronic and acute inflammatory cells, as well bacterial colonies (Figure 4).

In addition, the serum C-terminal cross-linking telopeptide of collagen (CTX) test to evaluate the bone reabsorption status was solicited and revealed normal values (250 pg/mL), but this exam was performed only 4 months after surgical treatment. The healing progressed uneventfully and the patient displayed no symptoms at 8 months of postoperative time.

**Figure 5:**
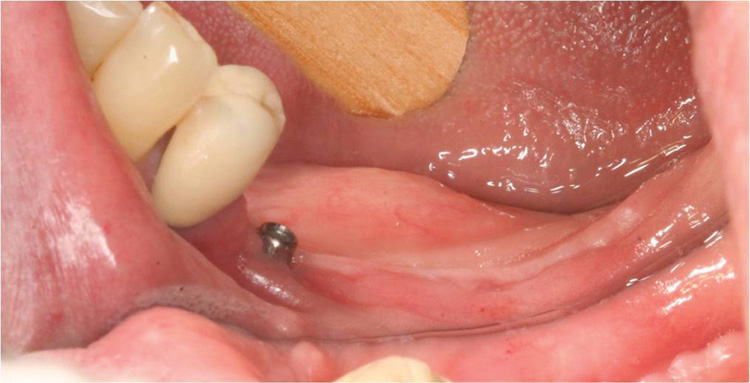
**Clinical aspects of the BRONJ lesions after treatment**. Post operatory of 9 month showing a mucosa with normal aspect without signals of inflammatory process or bone exposure

Observations in the clinical examination showed that the soft tissue was with normal aspect and without any signs of inflammatory or infectious processes (Figure 5)

## Case 2

A 68-year-old woman was admitted to a private dental clinic in October 2009, complaining about cold tooth sensation in the region of left mandibular second premolar. Review of the patient's medical history revealed that since 2003, she had been taking 2.5 mg of methotrexate six times a week and 70 mg of Fosamax^® ^(alendronate sodium) once a week for the treatment of rheumatoid arthritis. Besides, she also reported steroids use during twenty years. The patient had no history of radiotherapy, infectious process or trauma in the maxillofacial region but did have a history of smoking.

**Figure 6:**
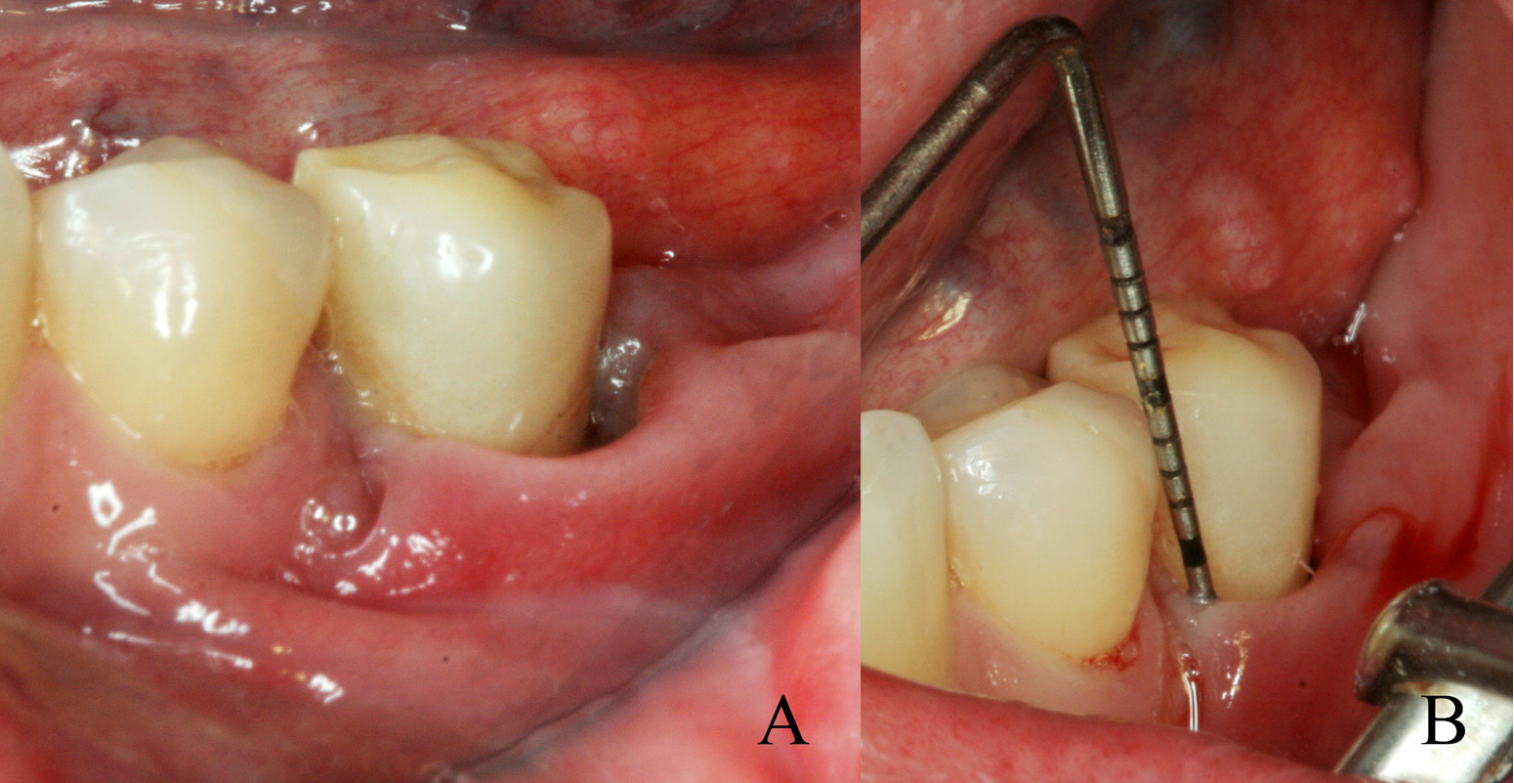
**Initial clinical aspects of the BRONJ lesion**. **A) **Detachment of the marginal gingival at the vestibular and distal side of # 35; **B) **Probing in the vestibular side of #35 showing increased probing depth values.

**Figure 7:**
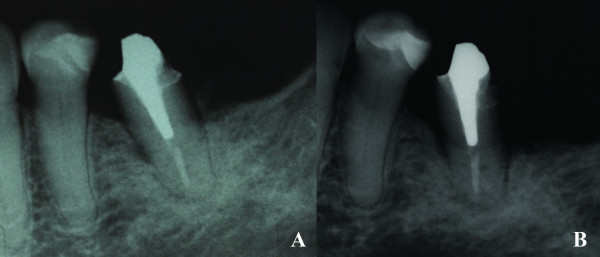
**Radiographic progression of bone loss in the BRONJ lesion**. **A) **Periapical radiographic showing bone loss associated with osteosclerosis around the #35; **B) **Periapical radiography showing increased bone loss around the #35.

During clinical examination a detachment of the marginal gingival (Figure 6a) associated with an increased probing depth value at the region of left mandibular second premolar (Figure 6b) was observed and was associated with a mild mobility without painful symptoms, purulent discharge and bone exposure. On periapical radiographic analysis, there was bone loss associated with osteosclerosis around the involved tooth (Figure 7a). At that time, mouth-rinsing with chlorhexidine 0.12% was prescribed and, after medical consensus, alendronate suspension was recommended. Furthermore, the serum C-terminal cross-linking telopeptide of collagen (CTX) test was solicited to evaluate the bone reabsorption status which revealed values of 33 pg/mL.

**Figure 8:**
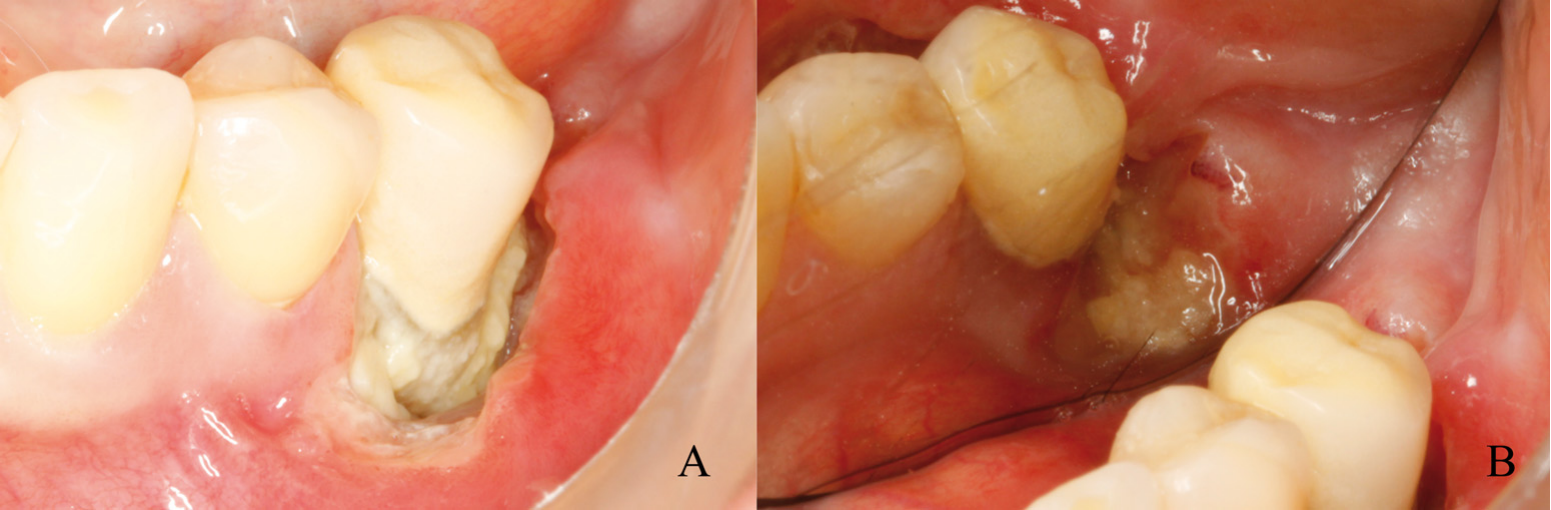
**Clinical progression of the BRONJ lesions**. **A) **Bone exposure of the #35 on the vestibular side; **B) **Bone exposure on the disto-lingual side of the edentulous alveolar bone surrounded by inflamed soft tissue.

**Figure 9:**
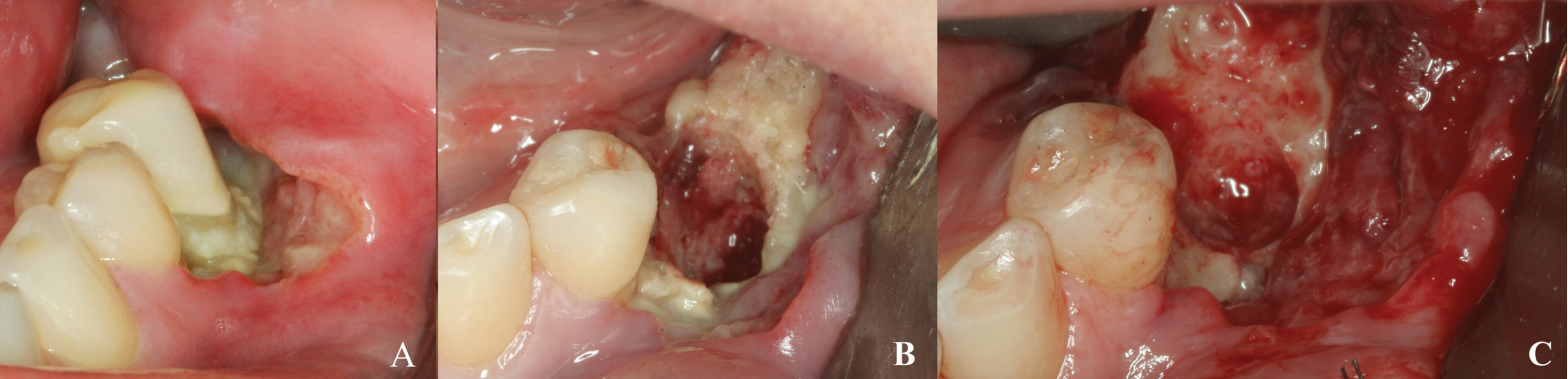
**Imaging aspect of the BRONJ lesion**. **A) **Computed tomography showing an irregular radiolucency at the left side of the mandible and a persistent alveolus of a molar that was extracted at least 10 years previously; **B**) Osteolysis around the left mandibular second premolar.

**Figure 10:**
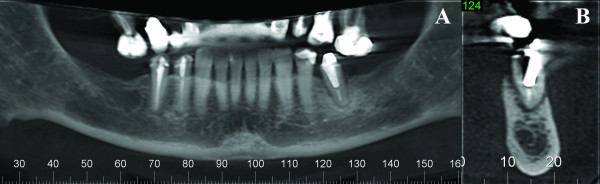
**Clinical progression of the BRONJ lesions**. **A) **Increasing of the bone necrosis around the #35 associated with a mucosal ulceration involving part of the jugal mucosa; **B) **Exposed bone area after the #35 extraction; **C) **Surgical area after bone debridement

Two weeks later, during clinical examination, bone exposure was detected on the vestibular side of the left mandibular second premolar and on the disto-lingual side of the edentulous alveolar bone surrounded by inflamed soft tissue without evidence of purulent discharge or pain symptoms (Figure 8). However, the lesions progressed very quickly and, the patient complained of painful symptoms and increased tooth mobility few days later. Bone necrosis associated with mucosa ulceration involving part of the jugal mucosa was also observed (Figure 9a). On periapical radiographic analysis, it was observed increased bone loss around the involved tooth (Figure 7b) which was confirmed on computed tomography (CT) since an osteolysis area was observed around the left mandibular second premolar associated with an intense bone sclerosis (Figure 10). Given these observations, a diagnosis of BRONJ could be established.

**Figure 11:**
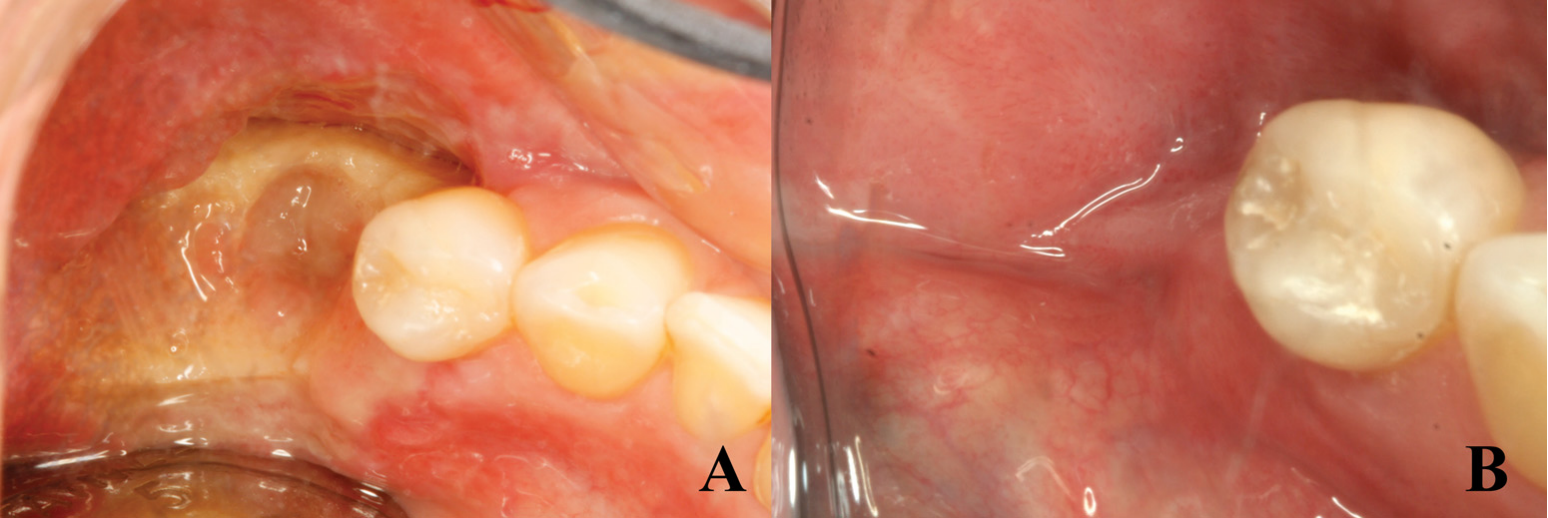
**Clinical aspects of lesions after 2 months of treatment**. **A) **Fourteen days after surgical debridement showing the formation of granulation tissue on the surgical area; **B) **Two months after surgical debridement showing a normal mucosa coverage of the involved area.

The management of the case included the tooth extraction and bone debridement under local anesthesia (Figure 9b and 9c), and mouth rinses with chlorhexidine plus antibiotic therapy with Clavulin 500 mg three times a day was prescribed. Within fourteen days, the formation of granulation tissue could be noted on the surgical area with no signs of inflammation or infection (Figure 11a). After two months, the debrided region was covered by normal mucosa with no painful symptoms (Figure 11b).

## Discussion

Rheumatoid arthritis is a systemic autoimmune disease characterized by progressive joint destruction and a variety of systemic manifestations resulting from chronic inflammation [3], which has been considered a risk factor for the development of BRONJ [1,2]. Although no scientific link has been established between BRONJ and RA, some relevant factors that could link these diseases should be discussed. These factors include inflammatory alterations and drugs prescribed for these patients, including steroids and immunosuppressive agents, such as methotrexate [4], that seem to play a relevant role in the development of oral BRONJ.

The relevance of steroids and methotrexate in BRONJ pathogenesis still remains not fully understood. However, considering that the main disease theories are based on the suppression of bone remodeling, the angiogenesis-inhibitory properties of the bisphosphonate and the infectious process [5] are factors that could be related to BRONJ; however, none of these theories have been completely accepted.

Hypothetical factors linked with BRONJ include a possible excessive suppression of bone turnover and jaw angiogenesis resulting from the association between bisphosphonates and steroids, since these drugs also reduce bone remodeling [6] and angiogenesis [7]. In addition, the immunosuppressive effects of steroids and methotrexate [8] could leave these patients more prone to infections.

In this discussion, observations that support and at the same time argue against this hypothetical association are made, especially in relation to steroid treatment. First of all, although a large number of patients with RA that develop oral BRONJ have a history of steroids and methotrexate intake [4,9-12] (as in case 2), this disease also occurs among patients with RA without the use of these drugs [9,13,14] (as in case 1). Second, it is well known that steroids can induce bone necrosis, but this necrosis differs from BRONJ because the steroids affect predominantly long bones and almost never produce bone exposure [15]. Finally, animal models of BRONJ have been proposed to test the association of bisphosphonate and steroids [16].

Recent tendencies included BPs among the most frequently prescribed drugs in rheumatologic practice [17] especially due to the high efficiency of BPs to be a protection against generalized bone loss [18]. In this way, patients with RA have been taking BPs to the prevention and treatment of osteoporosis which is a common feature in RA for several reasons including: post-menopausal women are the main risk group for RA and are at risk for accentuated bone loss; steroid therapy is often prescribed for the treatment of RA; physical inactivity is characteristic of RA due to disease activity; and bone loss due to disease inflammatory mechanisms, such as systemic elevated cytokines [19]. For these reasons, it is reasonable to believe that the incidence of BRONJ will increase as a result of the long-term use of BPs.

Regarding the link between inflammation and BRONJ, it is well known that extraarticular structures also can be affected by the inflammatory process in RA [20]. Considering that this disease is characterized by persistent high levels of proinflammatory cytokines [21] and accumulation of inflammatory cells [20], a link factor can be hypothesized based on the observations made by Lesclous et al. [22], who stated that BRONJ is associated with inflammation and that the clinical extension of the lesions is associated with the number of inflammatory cells.

According to the cases reported in literature, patients with RA who develop BRONJ lesions after oral administration of BPs are usually women, above 60 years old, who have taken alendronate for more than 3 years. The mandible is the most common site of BRONJ in these patients. The cases reported here are in agreement with this profile, except that the patient described in case 1 is younger than 60 years old. Pazianas et al. [23] have made the interesting observation that these features have exactly the same characteristics for patients without RA that develop oral BRONJ.

Most of the oral BRONJ cases in patients with or without RA are triggered by invasive dental procedures, such as extractions and dental implants. However, other cases of BRONJ can be spontaneous [1,10,12] as seen in the cases reported in the present paper. However, some concerns should be discussed. In case 1, although no apparent precipitant factor was present, trauma may have been a trigger event [24]. An eventual occlusal overload on the prosthesis might have contributed to BRONJ, because pain symptoms appeared soon after the prosthesis replacement.

Another relevant factor is seen in case 2. Although there was no previous dentistry procedure, the patient had periodontal disease. Periodontal disease has been considered by some authors to be a trigger event [25] due to the fact that this disease could increase the potential quantity of BPs released. However, this theory is still controversial [26]. An interesting observation is that individuals with rheumatoid arthritis are more likely to experience moderate to severe periodontal disease compared to their healthy counterparts [27]. This clinical association between the two diseases might be due to a common underlying pathobiology of periodontitis and rheumatoid arthritis [28].

The main clinical aspects of patients with RA who develop oral BRONJ include bone exposure, edema, pain and purulent discharge [9-11,13,29,30]. These features represent stage 2, as described by Ruggiero et al. [26], and indicate the lack of early attention to these patients in initial stages because these stages include nonspecific signals and symptoms in the oral cavity with no clinical evidence of bone exposure. In case 2, lesions progressed rapidly generating a great concern since in advanced stages of BRONJ lesions, paresthesia, fistula formation and pathologic fracture can also be present [9], although these features are more common in neoplasic patients [29].

According Ruggiero et al. [26], one of the diagnosis criteria of BRONJ is the presence of exposed bone in the maxillofacial region persisting for more than 8 weeks. Although most patients with RA have some kind of bone exposure, this BRONJ definition has been revised, due to some contrary observations. First, even advanced cases can also occur with no bone exposure in oral cavity [1]. Second, there is a lack of knowledge about early clinical features and their progression toward frank BRONJ [9]. This is well-illustrated in case 2, which shows the complete evolution of a BRONJ lesion in which it was possible to identify an early soft tissue necrosis and increased probing depth values that progressed to exposed bone area. Another concern about this case is that the distinction of early stages of BRONJ from other diagnoses, such as localized reacutization of chronic periodontitis, may be difficult [13].

The appropriate management of patients with BRONJ remains undefined and no widely accepted treatment protocol exists. Although it has been stated that surgical procedures may achieve better outcomes in non-neoplastic patients [29], Marx et al. [25] state that surgical procedures are not effective on patients with BRONJ and that these procedures lead to further exposed bone, worsening of the symptoms and a greater risk of pathologic fracture. These effects of surgery indicate long-term antibiotics and chlorhexidine 0.12% as treatment. The literature has shown that treatment of the lesions in patients with RA using this approach along with the discontinuation of the RA drugs have mostly positive outcomes, including the complete healing of the lesions [10,12,14]. In contrast, surgical therapy literature shows more divided outcomes, including both positive [1,30] and poor outcomes [9,4,24]. In the cases reported in this paper, surgical therapy was chosen, and excellent outcomes were achieved.

The assessment of the risk of BRONJ for patients taking BPs is a challenge. Marx et al. (2007) report use of C-terminal cross-linking telopeptide of type I collagen (CTX) test as an indicator of the risk of BRONJ, suggesting that values of less than 100 pg/mL represent a high risk and more than 150 pg/mL a low risk. In this report were found both normal values for CTX test (250 pg/mL in case 1) as abnormal values (33 pg/mL in case 2). However, the patient CTX test in case 2 would be normal if the scale purposed by Lehrer et al. (2008) is considered where values ranging 32 from 580 pg/ml are considered to be normal. Moreover, normal serum bone markers also can be found in patients with BRONJ still using BPs [31]. Other relevant point is that patient 1 just did the exam 4 month after the drug suspension and after surgical treatment, which may contributed for this normal values, as after the drug interruption there is a gradually improvement in the values of CTX test [10,31].

We acknowledge that a limitation of the present paper is the fact that it presents two BRONJ clinical cases in RA patients. Therefore, we cannot validate any hypothesis that could explain a definite association of synergistic actions of both RA and BRONJ. More studies should be developed with rigorous case ascertainment criteria, as well as appropriate documentation of risk factors and modifiers to support scientific bases for this hypothesis.

However, the present paper helps to highlight the need for a change in clinical practice or diagnostic/prognostic approaches related to BRONJ. Considering that BPs are among the most frequently prescribed drugs in rheumatologic practice [17], associated with the lack of knowledge about this disease among rheumatologists in many countries, it is reasonable to expect an increased tendency in the number of BRONJ reports involving RA patients. This fact shows the clear necessity for the improvement in the epidemiological vigilance systems of Public Health Entities, as well as a better coordination of safety-related pharmacovigilance initiatives.

## Conclusions

Although some features seem to link RA with oral BRONJ and act as synergistic effects, more studies should be developed to support the scientific bases for this hypothesis. In addition, most patients with RA and oral BRONJ are diagnosed in stage 2, which indicates the necessity for rheumatologists to be aware of the potential risk to their patients of developing BRONJ and to work together with dentists for the prevention and early detection of the lesions.

## Consent

Written informed consent was obtained from the patients for publication of these case reports and any accompanying images. A copy of the written consent form is available for review by the Editor-in-Chief of this journal.

## Competing interests

The authors declare that they have no competing interests.

## Authors' contributions

NCN performed one surgery under the supervison of the corresponding author, analyzed the records, reviewed all patients' data and designed the case report. ASB drafted the manuscript and helped in writing the text. LCS and RACM drafted the manuscript and reviewed it critically. EMJ performed one of the surgical procedures and reviewed the manuscript. All authors read and approved the final manuscript.

## Authors' Information

NCN is a PhD student from Implantology program at Araraquara School of Dentistry and ASB is a PhD student from Periodontology program at Araraquara School of Dentistry. LCS is a professor and the chairman of the Department of Physiology and Pathology, Division of Pathology at Araraquara School of Dentistry. EMJ and RACM are professors and chairmen of the Department of Diagnosis and Surgery, Division of Periodontology at Araraquara School of Dentistry.

## References

[B1] Park W Kim NK Kim MY Rhee YM Kim HJ Osteonecrosis of the jaw induced by oral administration of bisphosphonates in Asian population: five cases Osteoporos Int 2010 21 527 33 10.1007/s00198-009-0973-3 19484166

[B2] Malden N Beltes C Lopes V Dental extractions and bisphosphonates: the assessment, consent and management, a proposed algorithm Br Dent J 2009 206 93 8 10.1038/sj.bdj.2009.5 19165270

[B3] Alamanos Y Drosos AA Epidemiology of adult rheumatoid arthritis Autoimmun Rev 2005 4 130 6 10.1016/j.autrev.2004.09.002 15823498

[B4] Santos C Alegre C Osteonecrosis maxilar, bifosfonatos y artritis reumatoide Med Clin 2008 130 37 10.1157/13114546 18221649

[B5] Allen MR Burr DB The pathogenesis of bisphosphonate-related osteonecrosis of the jaw: so many hypotheses, so few data J Oral Maxillofac Surg 2009 67 61 70 19371816 10.1016/j.joms.2009.01.007

[B6] Patschan D Loddenkemper K Buttgereit F Molecular mechanisms of glucocorticoid induced osteoporosis Bone 2001 29 498 505 10.1016/S8756-3282(01)00610-X 11728918

[B7] Greenberger S Boscolo E Adini I Mulliken JB Bischoff J Corticosteroid suppression of VEGF-A in infantile hemangioma-derived stem cells N Engl J Med 2010 362 1005 13 10.1056/NEJMoa0903036 20237346 PMC2845924

[B8] Jain A Witbreuk M Ball C Nanchahal J Influence of steroids and methotrexate on wound complications after elective rheumatoid hand and wrist surgery J Hand Surg Am 2002 27 449 55 10.1053/jhsu.2002.32958 12015719

[B9] Yarom N Yahalom R Shoshani Y Hamed W Regev E Elad S Osteonecrosis of the jaw induced by orally administered bisphosphonates: incidence, clinical features, predisposing factors and treatment outcome Osteoporos Int 2007 18 1363 70 10.1007/s00198-007-0384-2 17598065

[B10] Marx RE Cillo JE Jr Ulloa JJ Oral bisphosphonate-induced osteonecrosis: risk factors, prediction of risk using serum CTX testing, prevention, and treatment J Oral Maxillofac Surg 2007 65 2397 410 10.1016/j.joms.2007.08.003 18022461

[B11] Barros SY Is your knowledge up-to-date? Bisphosphonate-related osteonecrosis of the jaw Int J Dent Hyg 2008 6 376 7 10.1111/j.1601-5037.2008.00342.x 19138190

[B12] Junquera L Gallego L Cuesta P Pelaz A de Vicente JC Clinical experiences with bisphosphonate-associated osteonecrosis of the jaws: analysis of 21 cases Am J Otolaryngol 2009 30 390 5 10.1016/j.amjoto.2008.07.014 19880027

[B13] Elad S Gomori MJ Ben-Ami N Friedlander-Barenboim S Regev E Bisphosphonate-related osteonecrosis of the jaw: clinical correlations with computerized tomography presentation Clin Oral Investig 2010 14 43 50 10.1007/s00784-009-0311-3 19603201

[B14] Lo JC O'Ryan FS Gordon NP Yang J Hui RL Prevalence of osteonecrosis of the jaw in patients with oral bisphosphonate exposure J Oral Maxillofac Surg 2010 68 243 53 10.1016/j.joms.2009.03.050 19772941 PMC10159647

[B15] Zigic TM Marcous C Hungerford DS Corticosteroid therapy associated with ischemic necrosis of bone in systemic lupus erythematosis Am J Med 1985 79 596 604 10.1016/0002-9343(85)90057-9 4061472

[B16] Sonis ST Watkins BA Lyng GD Lerman MA Anderson KC Bone changes in the jaws of rats treated with zoledronic acid and dexamethasone before dental extractions mimic bisphosphonate-related osteonecrosis in cancer patients Oral Oncol 2009 45 164 72 10.1016/j.oraloncology.2008.04.013 18715819

[B17] Yip RML Bisphosphonates and Osteonecrosis of the Jaw Hong Kong Bull Rheum Dis 2008 8 19 25

[B18] Breuil V Euller-Ziegler L Bisphosphonate therapy in rheumatoid arthritis Joint Bone Spine 2006 73 349 54 10.1016/j.jbspin.2005.10.019 16616575

[B19] Joffe I Epstein S Osteoporosis associated with rheumatoid arthritis: Pathogenesis and management Seminars in Arthritis and Rheumatism 1991 20 256 272 10.1016/0049-0172(91)90021-Q 2042057

[B20] Bartold PM Marshall RI Haynes DR Periodontitis and rheumatoid arthritis: a review J Periodontol 2005 76 2066 74 10.1902/jop.2005.76.11-S.2066 16277578

[B21] Snyderman R McCarty GA Genco RJ, Mergenhagen SE Analogous mechanisms of tissue destruction in rheumatoid arthritis and periodontal disease Host-Parasite Interaction in Periodontal Disease 1982 1 1 Washington, DC: American Society for Microbiology 354 362

[B22] Lesclous P Abi Najm S Carrel JP Baroukh B Lombardi T Bisphosphonate associated osteonecrosis of the jaw: a key role of inflammation? Bone 2009 45 843 52 10.1016/j.bone.2009.07.011 19631301

[B23] Pazianas M Miller P Blumentals WA Bernal M Kothawala P A review of the literature on osteonecrosis of the jaw in patients with osteoporosis treated with oral bisphosphonates: prevalence, risk factors, and clinical characteristics Clin Ther 2007 29 1548 58 10.1016/j.clinthera.2007.08.008 17919538

[B24] Sedghizadeh PP Stanley K Caligiuri M Hofkes S Lowry B Shuler CF Oral bisphosphonate use and the prevalence of osteonecrosis of the jaw: an institutional inquiry J Am Dent Assoc 2009 140 61 6 19119168 10.14219/jada.archive.2009.0019

[B25] Marx RE Sawatari Y Fortin M Broumand V Bisphosphonate-induced exposed bone (osteonecrosis/osteopetrosis) of the jaws: risk factors, recognition, prevention, and treatment J Oral Maxillofac Surg 2005 63 1567 75 10.1016/j.joms.2005.07.010 16243172

[B26] Ruggiero SL Dodson TB Assael LA Landesberg R Marx RE Mehrotra B American Association of Oral and Maxillofacial Surgeons J Oral Maxillofac Surg 2009 67 2 12 19371809 10.1016/j.joms.2009.01.009

[B27] Mercado F Marshall RI Klestov AC Bartold PM Is there a relationship between rheumatoid arthritis and periodontal disease? J Clin Periodontol 2000 27 267 72 10.1034/j.1600-051x.2000.027004267.x 10783841

[B28] Modi DK Chopra VS Bhau U Rheumatoid arthritis and periodontitis: biological links and the emergence of dual purpose therapies Indian J Dent Res 2009 20 86 90 10.4103/0970-9290.49070 19336867

[B29] Favia G Pilolli GP Maiorano E Osteonecrosis of the jaw correlated to bisphosphonate therapy in non-oncologic patients: clinicopathological features of 24 patients J Rheumatol 2009 36 2780 7 10.3899/jrheum.090455 19884275

[B30] Malden NJ Pai AY Oral bisphosphonate associated osteonecrosis of the jaws: three case reports Br Dent J 2007 203 93 7 10.1038/bdj.2007.636 17660780

[B31] Kunchur R Need A Hughes T Goss A Clinical investigation of C-terminal cross linking telopeptide test in prevention and management of bisphosphonate-associated osteonecrosis of the jaws J Oral Maxillofac Surg 2009 67 1167 73 10.1016/j.joms.2009.02.004 19446200

